# Effect of weekend admission on mortality associated with severe acute kidney injury in England: A propensity score matched, population-based study

**DOI:** 10.1371/journal.pone.0186048

**Published:** 2017-10-10

**Authors:** Nitin V. Kolhe, Richard J. Fluck, Maarten W. Taal

**Affiliations:** 1 Department of Renal Medicine, Royal Derby Hospital, Derby, United Kingdom; 2 Division of Medical Sciences and Graduate Entry Medicine, University of Nottingham, Derby, United Kingdom; Postgraduate Medical Institute, INDIA

## Abstract

**Background:**

Increased in-hospital mortality associated with weekend admission has been reported for many acute conditions, but no study has investigated “weekend effect” for acute kidney injury requiring dialysis (AKI-D).

**Methods:**

In this large, propensity score matched cohort of AKI-D, we examined the impact of weekend admission and in-centre nephrology services in 53,170 AKI-D admissions between 1^st^ April 2003 and 31^st^ March 2015 using a hospital episode statistic dataset. Propensity score matching (PSM) was performed to match 4284 weekend admissions with AKI-D with 14,788 admissions on weekdays.

**Results:**

Of the 53,170 admissions with AKI-D in the whole dataset, 12,357 (23%) were at weekends. The unadjusted mortality for weekend admissions was significantly higher compared to admissions on weekdays (40·6% versus 39·6%, p 0·046). However, in multivariable analysis of the PSM cohort, the odds of death for weekend admissions with AKI-D was 1·01 (95%CI 0·93,1·09). Mortality was higher for weekend admissions in West Midlands (odds ratio (OR) 1·32, 95% confidence interval (CI) 1·05, 1·66) and lower in East of England (OR 0·77, 95%CI 0·59, 1·00) but was not different to weekday admissions in all other regions. In 2003–04, weekend admissions had lower odds of death (OR 0·45, 95%CI 0·21, 0·96) and in 2010–11 higher odds of death (OR 1·28, 95%CI 1·00, 1·63) but in the other ten years observed, there was no significant difference in mortality between weekday and weekend admissions. Provision of in-centre nephrology services was associated with lower odds of death at 0·57 (95%CI 0·54, 0·62).

**Conclusions:**

Weekend admissions in patients with AKI-D had no effect on mortality. Further research is warranted to elucidate the reasons for the lower mortality in hospitals with in-centre nephrology services.

## Introduction

Several studies have reported an unwanted adverse effect of weekend admission on mortality in certain acute medical conditions [1]. Though the reason for this variation could be illness severity, some studies have hypothesized that the higher mortality could be due to variation in care provided over weekends when services are at a minimum [2]. The increased mortality over weekends for certain conditions in the National Health Service (NHS) in England has led to an intense debate on reconfiguring the health service [3]. Little is known about the impact of the weekend effect on severe acute kidney requiring dialysis (AKI-D) in England, the incidence of which has increased more than 12-fold over the past 15 years [4]. Most nephrology departments in England provide continuous consultant cover over weekends and have the capacity to perform emergency dialysis as needed over weekends suggesting that a weekend effect is less likely to occur. Patients admitted over the weekend may present in three ways—with severe AKI requiring dialysis over the weekend, with AKI and requiring dialysis on a subsequent weekday or with no AKI, but develop AKI-D during the in-patient stay over next few days. Some studies have reported that starting dialysis on Sunday or a diagnosis of severe AKI over weekends, does not affect mortality, while others have reported increased mortality for AKI in all sizes of hospital over weekends [5, 6]. One explanation for this observation is that necessary dialysis therapy may not be initiated on a weekend because of limited physician, nurse staff or device availability. However, bias can be created by single center studies as it is well recognized that the epidemiology of AKI-D shows considerable regional variation and larger studies are therefore required to adequately investigate this issue [7].

With this background, the objective of this study was to investigate whether a weekend effect on mortality exists for patients with AKI-D in England. We hypothesized that patients admitted over weekends would have increased mortality irrespective of demographic features and clinical characteristics. We also hypothesized that the effect on mortality would be more pronounced in centers with no on-site nephrology services.

## Material and methods

### Study design and patients

We extracted 2003–2015 data from the Hospital Episode Statistics (HES) database containing details of all admissions at National Health Service (NHS) hospitals in England [8]. We extracted each AKI inpatient stay from HES data as a finished discharge spell [9]. To reduce risk of bias we extracted only patients who had a discharge outcome in the HES database. Patients who did not have discharge outcome recorded were more likely to be categorized as alive, increasing the risk of bias. HES data provide a rich source of information and are increasingly being used for epidemiological studies [10–16].

We identified AKI cases by using validated International Classification of Diseases, Tenth Revision, Clinical Modification (ICD-10-CM) codes of N17 in any diagnosis codes. The ICD10 N17 codes for AKI have been previously been validated in the HES database [17, 18]. We extracted all available diagnosis codes and Office of Population Censuses and Surveys Classification of Interventions and Procedures, 4th revision (OPCS-4) codes. AKI-D was identified by OPCS code of X40.3 for hemodialysis or X40.4 for hemofiltration in any of the 24 procedures. To exclude patients starting maintenance dialysis, we excluded ICD10 codes N18.5 and N18.6 for chronic kidney disease stage five and end stage renal disease respectively and OPCS-4 codes for arteriovenous fistula (L74.2) or arteriovenous shunt (L74.3) during the inpatient admission. This algorithm has high positive and negative predictive values (all >90%) and has been used previously to identify patients with AKI-D [19]. The study protocol was examined by the Derby Hospital NHS foundation research and development department and the National Research Ethics Committee, East of England—Cambridge Central and was deemed exempt from ethical approval because the research involved non-identifiable information, previously collected in the course of normal care and available for public use.

### Measurements

The primary outcome variable was in-hospital mortality. The primary independent variable was day of hospital admission. Weekend admission was defined as admission on Saturday and Sunday. The Index of Multiple Deprivation is the official measure of relative deprivation (for neighborhoods) in England. The Index of Multiple Deprivation ranks every small area in England from one (most deprived area) to 32,844 (least deprived area) and deciles are calculated dividing the ranking into ten equal groups [20]. Data for hospitals which have onsite nephrology services with provision of acute dialysis were extracted from the United Kingdom Renal Registry (UKRR) website and was crosschecked with the website of each renal unit [21].

### Propensity score matching

To recapitulate the design of a randomized trial, patients were assigned a propensity score using a logistic regression model with day of admission as the outcome. Model selection was performed using stepwise regression using covariates, age, gender, AKI in diagnostic codes, admission method, ethnicity and the following comorbidities–acute myocardial infarction, congestive cardiac failure, peripheral vascular disease, peptic ulcer, dementia, diabetes, diabetic complications, liver disease, connective tissue disorders, pulmonary diseases, cirrhosis of liver, cancer, paraplegia, renal disease, metastatic disease and HIV status. We did not match covariates in each year because this would have resulted in too few cases for analysis. Matching was performed using a “one to many” technique where patients admitted on weekends were matched to many AKI-D patients who were admitted on weekdays and had the most similar estimated propensity score. To ensure good matches, a caliper (maximum allowable difference between two participants) of 0.053 was defined. Model adequacy checks were performed using standardized mean differences and the variance ratio in the group with weekend admission and weekday admission before and after matching

### Statistical analysis

Data during the 12-year period were stratified according to admission day either as weekday or weekend. The primary independent variable was admission on weekends (Saturday or Sunday) versus weekdays. We categorized age into four groups: <65, 65–74, 75–84 and>85 years. Charlson comorbidity index (CCI) was calculated and those with CCI greater than five were grouped into one category. Admission method was defined as one of elective admission; emergency admission; maternity admission; other admission or unknown. Ethnicity was grouped according to HES data dictionary as White, Mixed, Asian, Black, other ethnic groups and ethnicity not stated/unknown. AKI in diagnosis codes were categorized as ‘AKI in primary diagnosis code’, ‘AKI in secondary diagnosis code’ and ‘AKI in other diagnosis codes’. Continuous variables were expressed as mean with 95% confidence interval (CI); these were compared with the t-test under the Central Limit Theorem. Categorical variables were expressed as proportions and compared with the chi square test. Significant differences are indicated in the table with APA-style formatting using subscript letters and are calculated at the 0.05 significance level, with Bonferroni correction. The associations between discharge status and gender, age group, ethnicity, AKI in diagnoses codes, method of admission, CCI, day of admission and deprivation were analyzed initially using univariable logistic regression. Likelihood ratio tests were used to consider relative contributions of all these variables simultaneously in a multivariable logistic regression model. The final model included age groups, gender, ethnicity, AKI in diagnosis code, admission method, CCI, day of admission and deprivation. We used weekend admission as the reference, which enabled us to compare mortality to weekday admission. In subgroup analysis, we also evaluate whether there was change in mortality in weekend versus weekday admissions over the 12-year period and in the nine regions of England. Sensitivity of the findings was examined by performing the analysis by excluding patients normally resident outside of England. The analysis was repeated separately for all ethnicities and after excluding all patients where ethnicity was not known (see supplementary appendix). All analyses were performed using IBM SPSS Statistics for Windows, Version 22.0. Armonk, NY: IBM Corp. This study is registered with ClinicalTrials.gov, number NCT02947698

## Results

**Fig 1:**
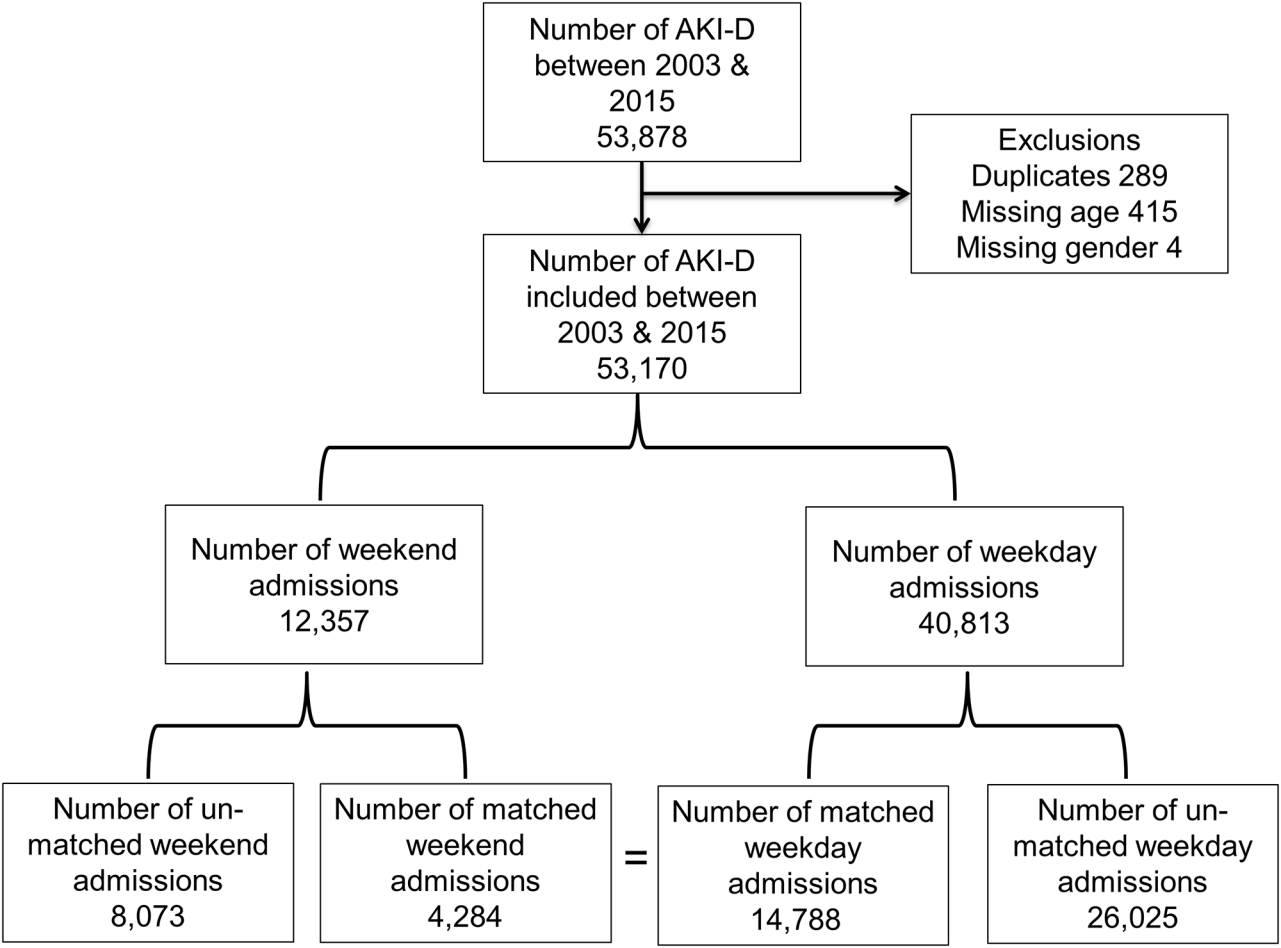
Study flow chart.

We identified 53,878 admissions with AKI-D in all hospitals in England between 1^st^ April 2003 and 31^st^ March 2015. Of these, 289 were duplicates and were excluded. Small numbers of patients with missing data items were also excluded (age in 415 and gender in 4) (Fig 1).

### Baseline characteristics of AKI-D patients before matching

Of the 53,170 admissions, 12,357 (23%) were during a weekend. Most characteristics of patients admitted over a weekend were similar to those admitted on weekdays (Table 1). During weekends, there was a greater proportion of emergency admissions (73.8% versus 70.7%, p <0.001) and transfers from one hospital to another (14.4% versus 12.7%, p < 0.001) and lower proportion of elective admissions (9.9% versus 14.4%) as compared to weekdays. Patients admitted on weekends were more likely to be from the most deprived (10%) category (8.5% versus 7.6%, p 0.036) and had lower prevalence of previous chronic kidney disease (35.9% versus 38.6, p < 0.001). The crude mortality for weekend admissions with AKI-D was marginally but significantly higher as compared to admissions on weekdays (40.6% versus 39.6%, p 0.046).

**Table 1:** Basic demography of admitted AKI-D patients in England grouped by day of admission between 2003–2015.

		Day of admission		p value
		Week days	Weekend	
Age group	<65 years	16141 (39.5)	4993 (40.4)	0.26
	65 to 74 years	11499 (28.2)	3394 (27.5)	
	75 to 84 years	11060 (27.1)	3352 (27.1)	
	≥ 85 years	2113 (5.2)	618 (5.0)	
Age in years^†^		64.8 ± 16.3	64.6 ± 16.4	0.148
Gender	Male	25477 (62.4)	7681 (62.2)	0.60
Ethnicity	White	32129 (78.7)	9691 (78.4)	0.62
	Mixed	185 (0.5)	64 (0.5)	
	Asian	2285 (5.6)	721 (5.8)	
	Black	1206 (3.0)	388 (3.1)	
	Any other ethnic group	693 (1.7)	215 (1.7)	
	Not known	4315 (10.6)	1278 (10.3)	
Admission method	Elective	5893 (14.4)	1219 (9.9)	<0.001
	Emergency	28864 (70.7)	9115 (73.8)	
	Maternity	27 (0.1)	7 (0.1)	
	Transfer	5198 (12.7)	1783 (14.4)	
	Not known	824 (2.0)	231 (1.9)	
AKI in diagnoses codes	Primary	15814 (38.7)	4682 (37.9)	0.20
	Secondary	6104 (15.0)	1851 (15.0)	
	All other codes	18895 (46.3)	5824 (47.1)	
Index of deprivation deciles	Most deprived	3108 (7.6)	1049 (8.5)	0.04
	2	3438 (8.4)	1066 (8.6)	
	3	3736 (9.2)	1102 (8.9)	
	4	3765 (9.2)	1178 (9.5)	
	5	4486 (11.0)	1305 (10.6)	
	6	4466 (10.9)	1322 (10.7)	
	7	4419 (10.8)	1371 (11.1)	
	8	4502 (11.0)	1291 (10.4)	
	9	4430 (10.9)	1358 (11.0)	
	Least deprived	4463 (10.9)	1315 (10.6)	
Acute Myocardial Infarction		5244 (12.8)	1635 (13.2)	0.27
Cerebrovascular accident		1876 (4.6)	618 (5.0)	0.06
Congestive Cardiac Failure		8334 (20.4)	2566 (20.8)	0.40
Connective Tissue Disorder		1141 (2.8)	335 (2.7)	0.62
Dementia		253 (0.6)	88 (0.7)	0.26
Peptic ulcer		836 (2.0)	247 (2.0)	0.73
Peripheral Vascular Disorder		3020 (7.4)	885 (7.2)	0.38
Lung disease		6264 (15.3)	1955 (15.8)	0.20
Paraplegia		336 (0.8)	98 (0.8)	0.74
Kidney disease		15746 (38.6)	4436 (35.9)	<0.001
Diabetes		11231 (27.5)	3413 (27.6)	0.82
Liver disease		3174 (7.8)	959 (7.8)	0.95
Malignancy		4344 (10.6)	1254 (10.1)	0.12
HIV		27 (0.1)	6 (0)	0.49
Mortality		39.6%	40.6%	0.046

^†^(mean ± Standard Deviation)

**Fig 2:**
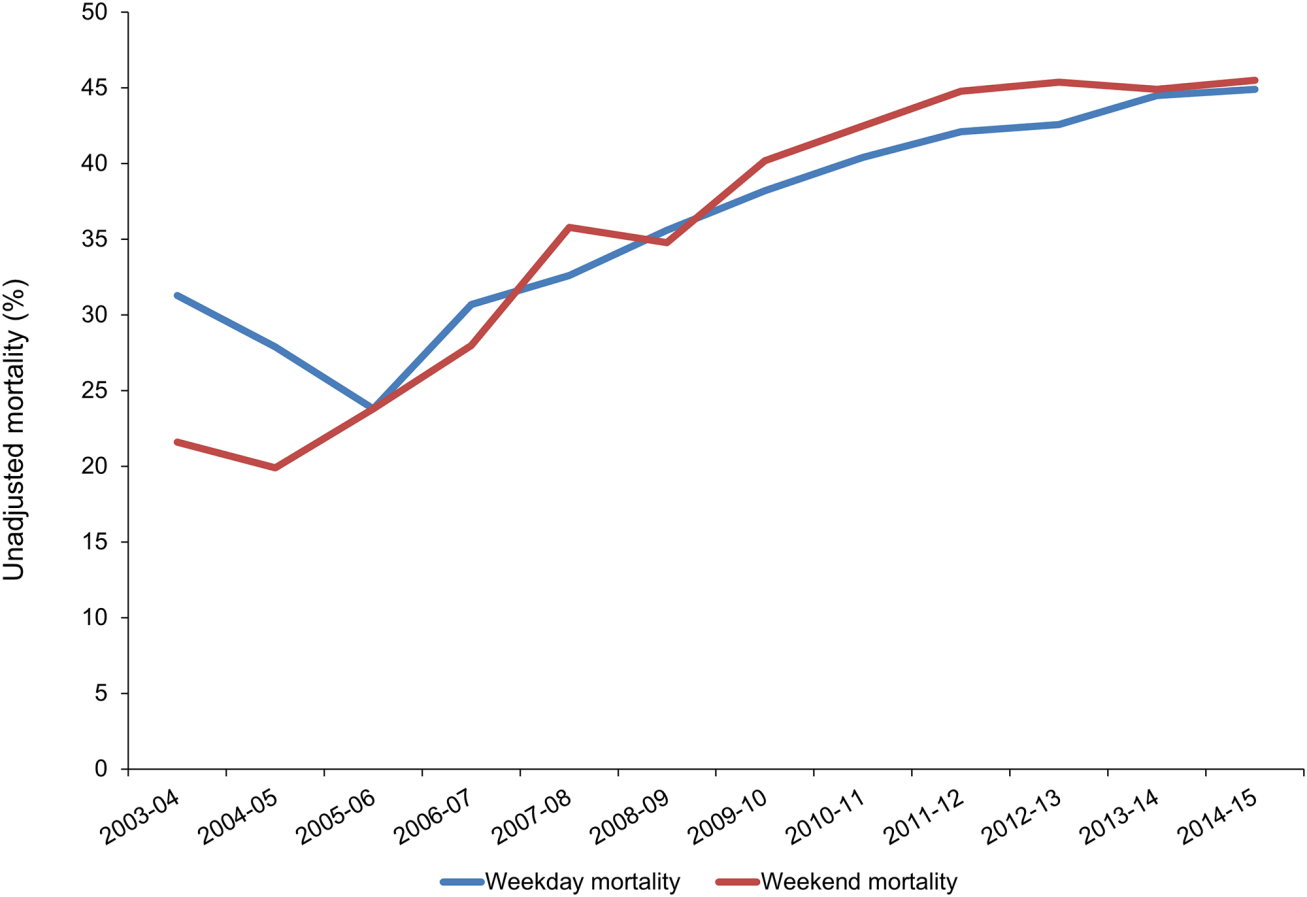
Unadjusted mortality by day of admission in each period over last fifteen years.

Crude mortality in patients admitted over weekends was lower in 2003–04 as compared to mortality in patients admitted on weekdays (21.6% versus 31.3%, p 0.002) (Fig 2). However, mortality increased over the next fifteen years and in 2014–15, it was higher for patients admitted over weekends as compared to those admitted on weekdays, though this was not statistically significant (45.5% versus 44.9%, p 0.6).

### Baseline characteristics of AKI-D patients after matching

Of the 12,537 admissions during weekends, 4284 were matched in a “one to many” technique with 14,788 admissions on weekdays. 26,025 weekday admissions and 8,073 weekend admissions were unmatched and not used. The standardised mean difference before matching was 0.157 and after matching was 0.053. Table 2 illustrates baseline characteristics of matched cohorts. All the baseline characteristics were well matched with no statistical difference. There was no statistical difference in the mortality between weekend and weekday admissions (38.2% versus 37.2%, p 0.3). We also compared baseline characteristics in each discharge period in the matched cohort (S1 Appendix).

**Table 2:** Basic demography of admitted AKI-D patients in England grouped by day of admission in a propensity score matched cohort between 2003–2015.

		Day of admission		p value
		Week days	Weekend	
Age group	<65 years	4477 (30.3)	1273 (29.7)	0.77
	65 to 74 years	5059 (34.2)	1460 (34.1)	
	75 to 84 years	4038 (27.3)	1203 (28.1)	
	≥85 years	1214 (8.2)	348 (8.1)	
Age in years^†^		67.9 ± 14.4	68.3 ± 14.1	0.169
Gender	Male	9279 (62.7)	2714 (63.4)	0.47
Ethnicity	White	11360 (76.8)	3234 (75.5)	0.23
	Mixed	25 (0.2)	3 (0.1)	
	Asian	906 (6.1)	272 (6.3)	
	Black	447 (3.0)	128 (3.0)	
	Any other ethnic group	230 (1.6)	72 (1.7)	
	Not known	1820 (12.3)	575 (13.4)	
Admission method	Elective	334 (2.3)	79 (1.8)	0.24
	Emergency	12649 (85.5)	3655 (85.3)	
	Maternity	25 (0.2)	7 (0.2)	
	Transfer	981 (6.6)	320 (7.5)	
	Not known	792 (5.4)	222 (5.2)	
AKI in diagnoses codes	Primary	7094 (48.0)	1992 (46.5)	0.07
	Secondary	2124 (14.4)	594 (13.9)	
	All other codes	5570 (37.7)	1698 (39.6)	
Index of deprivation deciles	Most deprived	1232 (8.3)	358 (8.4)	0.69
	2	1538 (10.4)	440 (10.3)	
	3	1544 (10.4)	471 (11.0)	
	4	1506 (10.2)	470 (11.0)	
	5	1562 (10.6)	417 (9.7)	
	6	1554 (10.5)	442 (10.3)	
	7	1562 (10.6)	452 (10.6)	
	8	1433 (9.7)	431 (10.1)	
	9	1429 (9.7)	407 (9.5)	
	Least deprived	1428 (9.7)	396 (9.2)	
Acute Myocardial Infarction		2015 (13.6)	604 (14.1)	0.43
Cerebrovascular accident		450 (3.0)	130 (3.0)	0.98
Congestive Cardiac Failure		3448 (23.3)	1030 (24.0)	0.32
Connective Tissue Disorder		720 (4.9)	202 (4.7)	0.68
Dementia		33 (0.2)	9 (0.2)	0.87
Peptic ulcer		483 (3.3)	132 (3.1)	0.55
Peripheral Vascular Disorder		1470 (9.9)	429 (10.0)	0.89
Lung disease		2189 (14.8)	630 (14.7)	0.88
Paraplegia		198 (1.3)	62 (1.4)	0.59
Kidney disease		12063 (81.6)	3497 (81.6)	0.93
Diabetes		4949 (33.5)	1455 (34.0)	0.54
Liver disease		1248 (8.4)	358 (8.4)	0.86
Malignancy		2152 (14.6)	616 (14.4)	0.78
HIV		17 (0.1)	3 (0.1)	0.42
Mortality		37.2%	38.2%	0.25

^†^(mean ± Standard Deviation)

### Effect of weekend admission, onsite nephrology services and deprivation on mortality in patients with AKI-D

**Fig 3:**
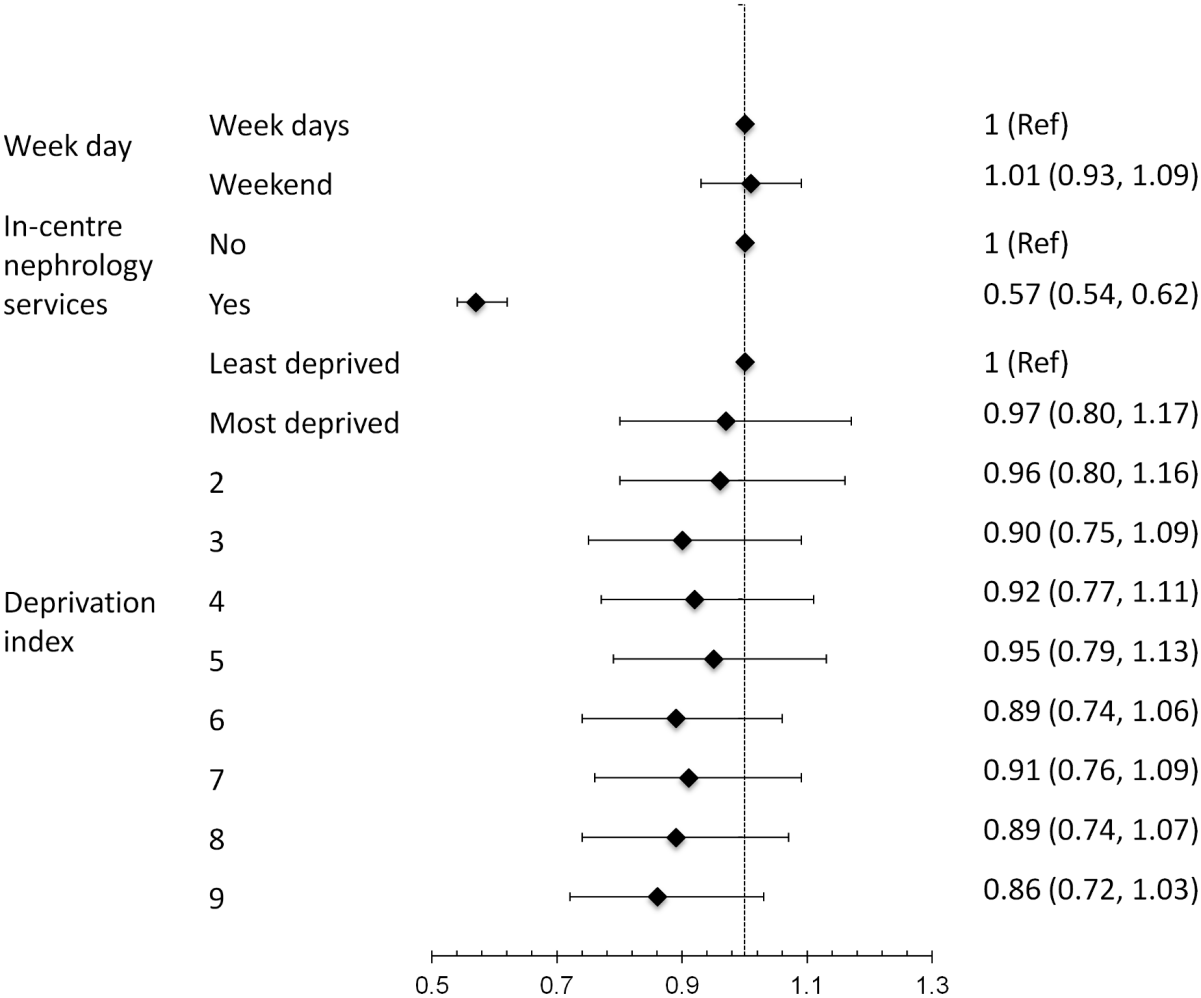
Multivariable adjusted odds ratios for death in patients who required dialysis for AKI.

In the propensity score matched cohort, the unadjusted for odds of death for weekend admission was not statistically significant at 1.04 (95% CI 0.96–1.12). After adjustment for all the above variables, the adjusted odds of death for admissions on weekend were similar at 1.01 (95%CI 0.93–1.09) (Fig 3). There was no effect of social deprivation on mortality due to AKI-D either for admissions on weekday or on weekend. Onsite nephrology services were associated with substantially lower odds of death (OR 0.57, 95% CI 0.54–0.62).

### Subgroup analysis

**Fig 4:**
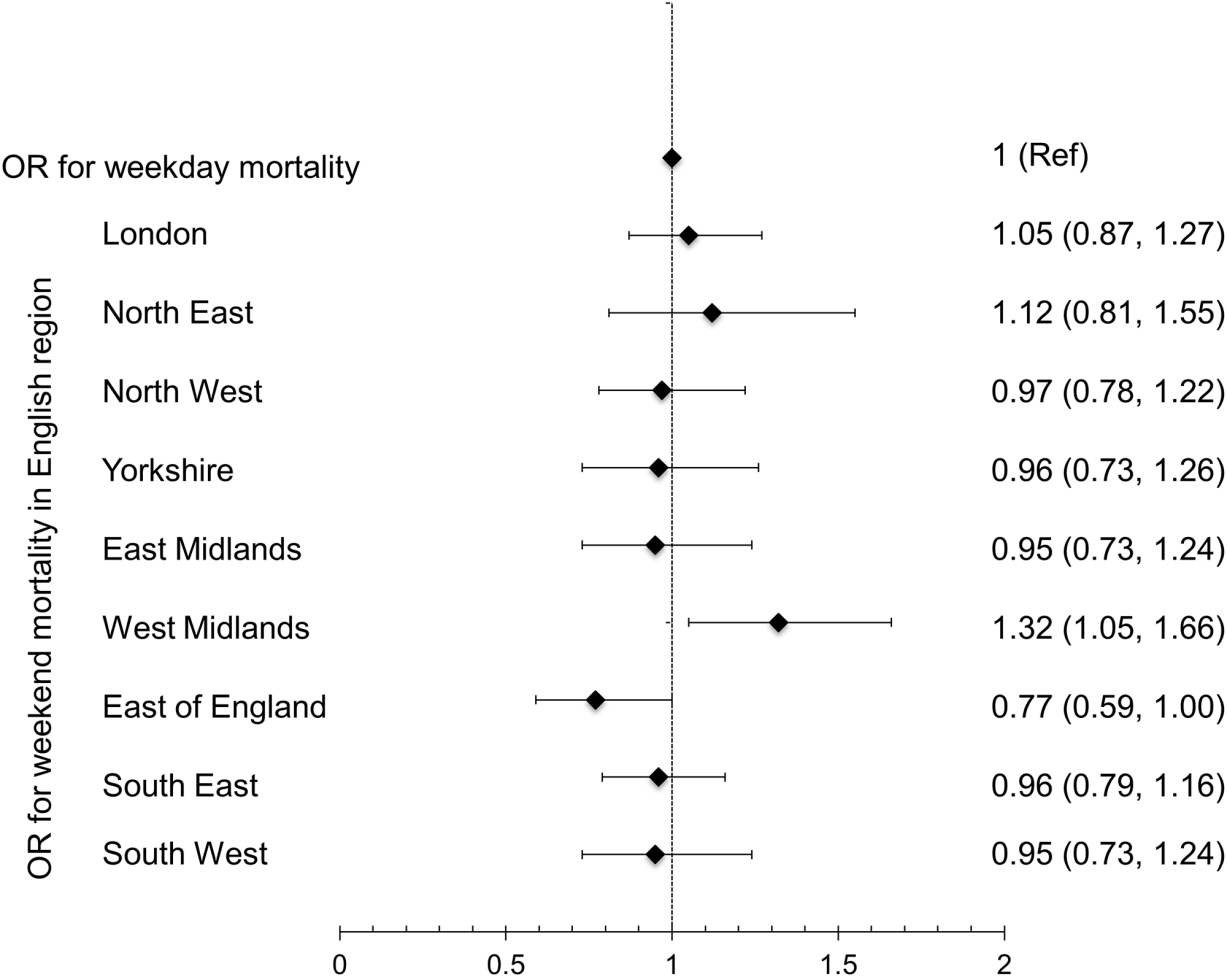
Adjusted mortality for weekend versus weekday admission in patients who required dialysis for AKI in each English region.

**Fig 5:**
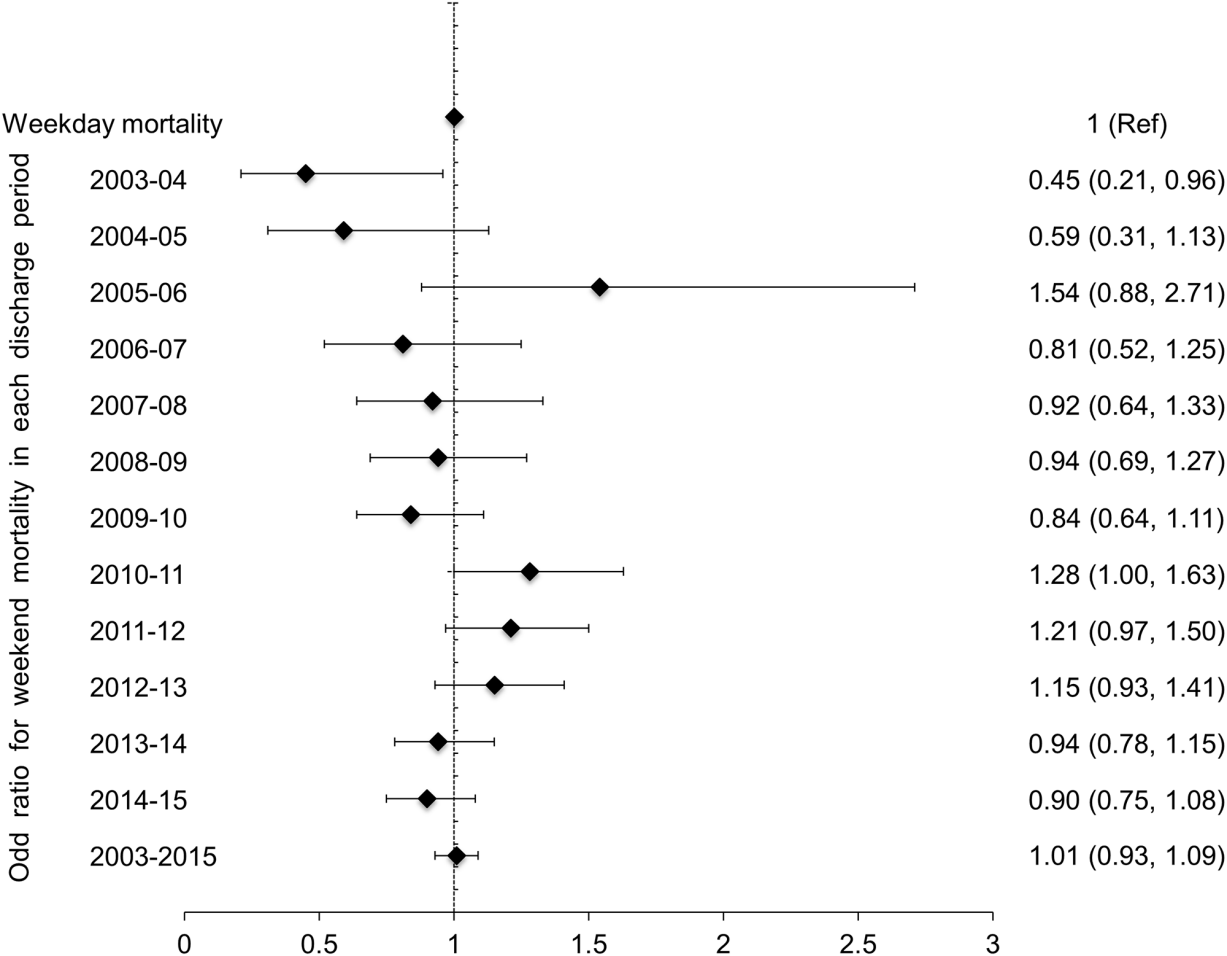
Adjusted mortality for weekend versus weekday admission in patients who required dialysis for AKI in each period of discharge studied.

In West Midlands, weekend admission was associated with higher odds of death (OR 1.32, 95%CI 1.05–1.66) and in East of England lowers odds of death (OR 0.77, 95%CI 0.59–1.00). In seven other regions, there was no significant weekend effect on mortality from AKI-D (Fig 4). In 2003–04, weekend admissions had lower odds of death (OR 0.45, 95% CI 0.21–0.96) and in 2010–11 the odds of death was higher (OR 1.28, 95% CI 1.00–1.63) with no significant difference in mortality between weekday and weekend admissions in the other ten years (Fig 5). The adjusted odds of death were significantly lower in hospitals with onsite nephrology services in all discharge periods from 2006–07 to 2014–15 and the odds ratio for the entire study period was 0.58 (95% CI 0.53–0.62) for weekday admissions and OR 0.57 (95% CI 0.50-.66) for weekend admissions (Table 3). The results of the two sensitivity analyses, after excluding patients with unknown ethnicity and patients normally residing outside England, were similar to the primary analysis (S1 and S2 Tables).

**Table 3:** Effect of onsite nephrology services on mortality in weekday and weekend admissions for AKI-D in each discharge period.

Discharge period	Weekday		Weekend	
	Univariate	Multivariable^§^	Univariate	Multivariable^§^
2003–04	0.58 (0.33, 1.05)	0.65 (0.33, 1.28)	0.29 (0.08, 1.08)	0.10 (0.01, 2.68)
2004–05	1.11 (0.61, 2.01)	1.34 (0.65, 2.72)	0.27 (0.09, 0.81)	0.07 (0.01, 0.57)
2005–06	0.75 (0.42, 1.36)	0.65 (0.32, 1.30)	1.35 (0.44, 4.14)	1.91 (0.27, 13.55)
2006–07	**0.59 (0.40, 0.87)**	0.70 (0.45, 1.10)	0.56 (0.26, 1.23)	0.48 (0.17, 1.34)
2007–08	**0.57 (0.41, 0.77)**	0.76 (0.53, 1.10)	**0.51 (0.28, 0.94)**	**0.42 (0.18, 0.95)**
2008–09	**0.40 (0.31, 0.53)**	**0.44 (0.32, 0.61)**	**0.44 (0.27, 0.72)**	**0.41 (0.23, 0.74)**
2009–10	**0.47 (0.37, 0.59)**	**0.47 (0.36, 0.61)**	0.76 (0.48, 1.20)	1.06 (0.60, 1.88)
2010–11	**0.51 (0.41, 0.63)**	**0.61 (0.48, 0.77)**	**0.47 (0.32, 0.69)**	**0.54 (0.34, 0.84)**
2011–12	**0.49 (0.40, 0.60)**	**0.52 (0.42, 0.65)**	**0.37 (0.26, 0.53)**	**0.39 (0.25, 0.59)**
2012–13	**0.45 (0.37, 0.54)**	**0.47 (0.38, 0.57)**	**0.42 (0.30, 0.59)**	**0.50 (0.33, 0.75)**
2013–14	**0.47 (0.39, 0.56)**	**0.57 (0.47, 0.70)**	**0.50 (0.36, 0.69)**	**0.60 (0.41, 0.88)**
2014–15	**0.58 (0.49, 0.68)**	**0.68 (0.56, 0.82)**	**0.53 (0.39, 0.72)**	**0.67 (0.46, 0.96)**
Overall 2003–15	**0.50 (0.47, 0.54)**	**0.58 (0.53, 0.62)**	**0.47 (0.52, 0.54)**	**0.57 (0.49, 0.66)**

Data are odds ratio (95% CI)

^**§**^ Adjusted for age group, gender, ethnicity, AKI in diagnoses codes, deprivation, admission methods, CCI

## Discussion

AKI is increasingly recognized as a severe illness associated with a high mortality. The incidence of AKI is increasing and though the associated mortality has decreased in the United States, this has not been the trend in England, especially for AKI-D [4, 22–24]. One reason postulated for the high mortality in England, is the “weekend effect”. However, in this propensity score matched analysis of routinely collected data from England’s national database, we did not observe increased mortality in AKI-D patients admitted on weekends between 2003 and 2015. There was no significant increase in odds of death in any of the discharge periods except 2010 to 2011 and there was no increase in mortality for weekend admissions with AKI-D in any regions except West Midlands over the study period. The provision of in-center nephrology services was independently associated with lower odds of death both for weekday and weekend admissions.

In a recent study, which included AKI patients with AKIN stage 2 or greater, Wilson et al found that, though the rate of starting dialysis on Sunday was lower than on weekdays, the inpatient mortality was similar for admissions on weekends and weekdays [5]. The authors suggested that lack of nursing and physician personnel on weekends accounted for the lower rate of dialysis on Sundays. Inspite of this, the study did not show increased mortality on Mondays, as might be expected if dialysis was deferred from Sunday to Monday. However, the study was from single health network, which could have created bias. In addition, the majority of AKI patients in the study did not need dialysis. In a recent study from England and Wales, the authors compared 30-day mortality for admissions on weekend and weekdays in 15 major disease groups [25]. Though the odds of death was higher for weekend admissions, this was not observed in all conditions. The weekend effect was more pronounced for abdominal aortic aneurysm and other disorders with very high mortality during the acute phase–pulmonary embolism, stroke, and subarachnoid hemorrhage. Acute myocardial infarction and less acute disorders like chronic obstructive pulmonary disease, pneumonia, hip fracture, acute pancreatitis, and inflammatory bowel disease showed little or no weekend effect. However, mortality for AKI was not studied. James et al studied AKI patients admitted on weekends between 2003 and 2006 [6]. They used ICD10 codes in primary and secondary diagnosis to identify AKI and found that weekend admission for AKI was associated with 7% increased odds for death as compared to weekday admission. They also found that smaller hospitals had increased odds for death as compared to medium and larger sized hospitals. However, there were significant differences between the patient characteristics admitted on weekday and weekend. In addition, admission method (elective versus emergency), which is known to affect mortality, was not stated for the two groups. The study predominantly had non-dialysis requiring AKI with less than 9% having AKI-D. Moreover, patients transferred from other acute care hospitals were excluded. Transfers between acute hospitals in England are common, as, not all hospitals have nephrology or acute dialysis facilities. Delay in nephrology consultation has previously been shown to be associated with increased ICU mortality in AKI patients [26]. This may also be the reason for lower odds of death in centers with onsite nephrology and acute dialysis services in our study. This is in keeping with a previous study demonstrating higher odds of death in AKI-D patients when they are transferred from one hospital to other [4].

Various reasons have been postulated for increased mortality on weekends for many disease groups. Lower levels of staffing and non-availability of diagnostic or therapeutic interventions are commonly cited reasons [2, 3, 27]. Over the weekend, routine services are reduced from Friday evening to Monday morning. However, this varies across different specialties. Continuous renal replacement therapy is available in most intensive care units in NHS hospitals, though a nephrologist may not always be involved in prescribing it. In this study, the availability of acute dialysis does not seem to have been a factor affecting weekend mortality but availability of in-center nephrology services was associated with lower mortality on both weekdays and weekends. Given the design of our study we were unable to elucidate the reason for increased mortality in centers with no in-center nephrology services. The weekend effect may vary in different healthcare settings, making comparison of studies difficult. The NHS is a single payer, publicly funded healthcare system providing healthcare based on need, and free at the point of use. A recent point prevalence survey of English NHS hospital trusts compared weekend to weekday admission risk of mortality with the Sunday versus Wednesday specialist staffing ratio within each Trust. Though the mortality risk in patients admitted at weekends was higher than those admitted on weekdays, the study did not detect a correlation between weekend staffing by hospital specialists and mortality risk for emergency admissions [28].

### Strengths and limitations of the study

The major strength of this study is the propensity score matching of case-mix by matching several important baseline characteristics, which are known to influence mortality, thus removing a major source of bias. The analysis in this study covers all AKI-D patients admitted in English NHS hospital over 12 years and therefore is a unique population-wide assessment. Using national data over a long time-period allows accounting for variation in hazard observed across the years, and provides a large number of events and patients at risk for analysis. It is also unlikely to be biased due to variation in funding between hospitals, insurance coverage or patients’ ability to pay.

This study is not without its limitations. First, we have used ICD-10 codes to identify AKI. Nevertheless, the algorithm we used to identify AKI-D has been shown previously to have good sensitivity and specificity and has been used in many epidemiological studies of AKI-D [4, 17, 18, 22]. In addition, it is highly unlikely that coding bias could have affected the results, as the coding procedure is the same for all admissions. Second, though we have attempted to control known confounders, there may be many other unmeasured confounders, which have not been taken into account. Third, the design of our study precluded us from investigating the reason for increased mortality in centers with no in-center nephrology services. Fourth, we did not match confounders in each year of discharge period as this would have resulted in too few cases to conduct a robust analysis. Nevertheless due to close matching of covariates we are confident that this did not affect the results with respect to the primary outcome.

In conclusion, patients admitted to NHS hospitals in England on weekends who develop severe AKI-D, did not have increased mortality as compared to patients admitted on weekdays. However, AKI-D patients had higher mortality when admitted to centers with no onsite nephrology services. Further studies are required to evaluate the relationship between onsite nephrology services and AKI-D mortality.

## Supporting information

S1 Appendix Confounders in propensity score matched cohort in each discharge period. (**Table A)**: Basic demography of admitted AKI-D patients in England grouped by day of admission in a propensity score matched cohort in each study period from 2003 to 2006.; (**Table B)**: Basic demography of admitted AKI-D patients in England grouped by day of admission in a propensity score matched cohort in each study period from 2006 to 2009.; (**Table C):** Basic demography of admitted AKI-D patients in England grouped by day of admission in a propensity score matched cohort in each study period from 2009 to 20012.; (**Table D):** Basic demography of admitted AKI-D patients in England grouped by day of admission in a propensity score matched cohort in each study period from 2012 to 2015.
(DOCX)

S2 Appendix STROBE checklist. (DOCX)

S1 Table Effect of weekend admission on mortality after exclusion of patients with unknown ethnicity. (DOCX)

S2 Table Effect of weekend admission on mortality after exclusion of patients normally resident outside of England. (DOCX)
