# Supplementary material for: Effect of weekend admission on mortality associated with severe acute kidney injury in England: A propensity score matched, population-based study
Source: PLoS One. 2017 Oct 10;12(10):e0186048. doi: 10.1371/journal.pone.0186048 (PMC5634642; doi:10.1371/journal.pone.0186048)
Supplement: S1 Appendix — (Table A): Basic demography of admitted AKI-D patients in England grouped by day of admission in a propensity score matched cohort in each study period from 2003 to 2006.; (Table B): Basic demography of admitted AKI-D patients in England grouped by day of admission in a propensity score matched cohort in each study period from 2006 to 2009.; (Table C): Basic demography of admitted AKI-D patients in England grouped by day of admission in a propensity score matched cohort in each study period from 2009 to 20012.; (Table D): Basic demography of admitted AKI-D patients in England grouped by day of admission in a propensity score matched cohort in each study period from 2012 to 2015. (DOCX) [file pone.0186048.s001.docx]

**S1 Appendix: Confounders in propensity score matched cohort in each discharge period**

In unadjusted analysis, all the baseline characteristics were well matched in seven of the twelve years, with differences in AKI codes in 2010-11 and 2012-13, connective tissue disorder in 2011-12 and 2013-14 and acute myocardial infarction in 2009-10. The unadjusted mortality was lower for weekend admission in 2003-04 (36.2% versus 22.4%, p 0.047 and higher in 2010-11 (36.6% versus 44.1%, p 0.004)

Table A: Basic demography of admitted AKI-D patients in England grouped by day of admission in a propensity score matched cohort in each study period from 2003 to 2006

|  |  | **2003-04** | | | **2004-05** | | | **2005-06** | | |
| --- | --- | --- | --- | --- | --- | --- | --- | --- | --- | --- |
|  |  | **Weekday** | **Weekend** | **p value** | **Weekday** | **Weekend** | **p value** | **Weekday** | **Weekend** | **p value** |
| Age group | <65 | 77 (30) | 17 (29.3) | 0.595 | 95 (30.2) | 33 (37.9) | 0.526 | 106 (29.3) | 32 (31.4) | 0.718 |
|  | 65 to 74 | 106 (41.2) | 22 (37.9) |  | 114 (36.2) | 27 (31) |  | 141 (39) | 43 (42.2) |  |
|  | 75 to 84 | 63 (24.5 | 14 (24.1) |  | 80 (25.4) | 19 (21.8) |  | 91 (25.1) | 20 (19.6) |  |
|  | >=85 | 11 (4.3) | x |  | 26 (8.3 | 8 (9.2) |  | 24 (6.6) | 7 (6.9) |  |
| Gender | Male | 159 (61.9( | 41 (70.7) | 0.23 | 185 (58.7) | 53 (60.9) | 0.713 | 223 (61.6) | 72 (70.6) | 0.104 |
| Ethnicity | White | 183 (7.2) | 36 (62.1) | 0.284 | 215 (68.3) | 59 (67.8) | 0.236 | 262 (72.4) | 72 (70.6) | 0.889 |
|  | Black | 10 (3.9) | x |  | 7 (2.2) | x |  | 12 (3.3) | x |  |
|  | Asian | 8 (3.1) | x |  | 7 (2.2) | 6 (6.9) |  | 14 (3.9) | 6 (5.9) |  |
|  | Not known | 55 (21.4) | 16 (27.6) |  | 83 (26.3) | 20 (23) |  | 71 (19.6) | 21 (20.6) |  |
| Admission method | Elective | 8 (3.1) | x | 0.191 | 7 (2.2) | x | 0.101 | 7 (1.9) | x | 0.28 |
|  | Emergency | 222 (86.4) | 47 (81) |  | 265 (84.1) | 79 (90.8) |  | 303 (83.7) | 78 (76.5) |  |
|  | Transfer | 26 (10.1) | 7 (12.1) |  | 42 (13.3) | 7 (8) |  | 50 (13.8) | 21 (20.6) |  |
|  | Not known | x | x |  | xx | 19 (21.8) |  | x | x |  |
| AKI in codes | Primary | 173 (67.3) | 40 (69) | 0.949 | 225 (71.4) | 59 (67.8) | 0.456 | 245 (67.7) | 69 (67.6) | 0.996 |
|  | Secondary | 35 (13.6) | 7 (12.1) |  | 32 (10.2) | 13 (14.9) |  | 40 (11) | 11 (10.8) |  |
|  | Other | 49 (19.1) | 11(19) |  | 58 (18.4) | 15 (17.2) |  | 77 (21.3) | 22 (21.6) |  |
| Comorbidities | Acute myocardial infarction | 31 (12.1) | 8 (13.8) | 0.43 | 31 (9.8) | 7 (8) | 0.685 | 32 (8.8) | 5 (4.9) | 0.221 |
|  | Cerebrovascular accident | 9 (3.5) | x | 0.695 | 7 (2.2) | x | 1 | x | x | 0.182 |
|  | Congestive cardiac failure | 49 (19.1) | 6 (10.3) | 0.129 | 37 (11.7) | 12 (13.8) | 0.583 | 48 (13.3) | 15 (14.7) | 0.744 |
|  | Connective tissue disorder | 10 (3.9) | x | 1 | 12 (3.8) |  | 0.758 | 14 (3.9) | x | 1 |
|  | Kidney disease | 205 (79.8) | 43 (74.1) | 0.375 | 254 (80.6) | 74 (85.1) | 0.435 | 305 (84.3) | 81 (79.4) | 0.293 |
|  | Peptic ulcer | 16 (6.2) | x | 0.329 | 14 (4.4) | x | 1 | 8 (2.2) | 6 (5.9) | 0.092 |
|  | Peripheral vascular disease | 25 (9.7) | x | 1 | 23 (7.3) | 8 (9.2) | 0.649 | 29 (8) | 13 (12.7) | 0.17 |
|  | Lung disease | 21 (8.2) | x | 0.588 | 28 (8.9) | 8 (9.2) | 1 | 39 (10.8) | 14 (13.7) | 0.384 |
|  | Paraplegia |  | x | x | x | x | x | 6 (1.7) | x | 0.69 |
|  | Diabetes mellitus | 57 (22.2) | 15 (25.9) | 0.604 | 77 (24.4) | 26 (29.9) | 0.332 | 116 (32) | 32 (31.4) | 1 |
|  | Liver disease | 9 (3.5) |  | 0.695 | 12 (3.8) |  | 0.743 | 15 (4.1) | x | 0.783 |
|  | Malignancy | 43 (16.7) | 15 (25.9) | 0.132 | 53 (16.8) | 8 (9.2) | 0.092 | 53 (14.6) | 15 (14.7) | 1 |
| Deprivation | Most deprived | 60 (23.3) | 20 (34.5) | 0.58 | 13 (4.1) | 6 (6.9) | 0.207 | 26 (7.2) | 10 (9.8) | 0.156 |
|  | Least deprived |  |  |  | 26 (8.3) | 11 (12.6) |  | 36 (9.9) | 15 (14.7) |  |
| Mortality |  | **93 (36.2)** | **13 (22.4)** | **0.047** | 92 (29.2) | 19 (21.8) | 0.222 | 93 (25.7) | 32 (31.4) | 0.258 |

Small numbers have been suppressed as part of disclosure control and indicated by x. Proportions in bold indicate statistical significance

Table B: Basic demography of admitted AKI-D patients in England grouped by day of admission in a propensity score matched cohort in each study period from 2006 to 2009.

|  |  | **2006-07** | | | **2007-08** | | | **2008-09** | | |
| --- | --- | --- | --- | --- | --- | --- | --- | --- | --- | --- |
|  |  | **Weekday** | **Weekend** | **p value** | **Weekday** | **Weekend** | **p value** | **Weekday** | **Weekend** | **p value** |
| Age group | <65 | 165 (30) | 45 (31) | 0.471 | 260 (31.5) | 74 (33.8) | 0.709 | 342 (31.8) | 97 (31) | 0.744 |
|  | 65 to 74 | 21 (38.2) | 49 (33.8) |  | 277 (33.5) | 76 (34.7) |  | 362 (33.6) | 115 (36.7) |  |
|  | 75 to 84 | 130 (23.6) | 42 (29) |  | 244 (29.5) | 56 (25.6) |  | 286 (26.6) | 79 (25.2) |  |
|  | >=85 | 45 (8.2) | 9 (6.2) |  | 45 (5.4) | 13 (5.9) |  | 87 (8.1) | 22 (7) |  |
| Gender | Male | 347 (63.1) | 95 (65.5) | 0.628 | 520 (63) | 132 (60.3) | 0.481 | 693 (64.3) | 189 (60.4) | 0.206 |
| Ethnicity | White | 382 (69.5) | 103 (71) | 0.893 | 618 (74.8) | 160 (73.1) | 0.483 | 805 (74.7) | 230 (73.5) | 0.614 |
|  | Black | 17 (3.1) | 6 (4.1) |  | 31 (3.8) | 6 (2.7) |  | 31 (2.9) | 11 (3.5) |  |
|  | Asian | 37 (6.7) | 7 (4.8) |  | 44 (5.3) | 9 (4.1) |  | 56 (5.2) | 22 (7) |  |
|  | Any other | x | x |  | 11 (1.3) | x |  | 26 (2.4) | x |  |
|  | Not known | x | x |  | x | X |  | 158 (14.7) | 46 (14.7) |  |
| Admission method | Elective | 11 (2) | x | 0.557 | 22 (2.7) | x | 0.314 | 21 (1.9) | 11 (3.5) | 0.519 |
|  | Emergency | 476 (86.5) | 123 (84.8) |  | 715 (86.6) | 186 (85.8) |  | 964 (89.5) | 277 (88.5) |  |
|  | Transfer | 59 (10.7) | 20 (13.8) |  | 84 (10.2) | 29 (13.2) |  | 86 (8) | 24 (7.7) |  |
|  | Not known | x | x |  | x | x |  | 12 | x |  |
| AKI in codes | Primary | 336 (61.1) | 87 (60) | 0.548 | 498 (60.3) | 129 (58.9) | 0.435 | 583 (54.1) | 178 (56.9) | 0.550 |
|  | Secondary | 62 (11.3) | 21 (14.5) |  | 102 (12.3) | 22 (10) |  | 143 (13.3) | 35 (11.2) |  |
|  | Other | 152 (27.6) | 37 (25.5) |  | 226 (27.4) | 68 (31.1) |  | 351 (32.6) | 100 (31.9) |  |
| Comorbidities | Acute myocardial infarction | 72 (13.1) | 18 (12.4) | 0.89 | 119 (14.4) | 23 (10.5) | 0.15 | 148 (13.7) | 40 (12.8) | 0.708 |
|  | Cerebrovascular accident | 11 (2) | x | 0.476 | 24 (2.9) | x | 0.817 | 28 (2.6) | 7 (2.2) | 0.839 |
|  | Congestive cardiac failure | 82 (14.9) | 30 (20.7) | 0.099 | 153 (18.5) | 49 (22.4) | 0.211 | 204 (18.9) | 48 (15.3) | 0.157 |
|  | Connective tissue disorder | 27 (4.9) | x | 0.655 | 30 (3.6) | x | 0.282 | 41 (3.8) | 14 (4.5) | 0.621 |
|  | Kidney disease | 441 (80.2) | 119 (82.1) | 0.639 | 703 (85.1) | 189 (86.3) | 0.747 | 912 (84.7) | 258 (82.4) | 0.334 |
|  | Peptic ulcer | 17 (3.1) | 6 (4.1) | 0.600 | 32 (3.9) | 10 (4.6) | 0.698 | 39 (3.6) | 10 (3.2) | 0.862 |
|  | Peripheral vascular disease | 55 (10) | 12 (8.3) | 0.636 | 71 (8.6) | 20 (9.1) | 0.788 | 99 (9.2) | 31 (9.9) | 0.741 |
|  | Lung disease | 36 (6.5) | 11 (7.6) | 0.71 | 89 (10.8) | 23 (10.5) | 1 | 130 (12.1) | 46 (14.7) | 0.246 |
|  | Paraplegia | 14 (2.5) | x | 1 | 9 (1.1) | x | 0.489 | 15 (1.4) |  | 1 |
|  | Diabetes mellitus | 166 (30.2) | 45 (31) | 0.84 | 286 (34.6) | 64 (29.2) | 0.147 | 339 (31.5) | 114 (36.4) | 0.101 |
|  | Liver disease | 21 (3.8) | x | 1 | 42 (5.1) | 14 (6.4) | 0.499 | 67 (6.2) | 18 (5.8) | 0.893 |
|  | Malignancy | 79 (14.4) | 25 (17.2) | 0.432 | 105 (12.7) | 31 (14.2) | 0.573 | 149 (13.8) | 33 (10.5) | 0.153 |
| Deprivation | Most deprived | 33 (6) | 9 (6.2) | 0.594 | 48 (5.8) | 9 (4.1) | 0.768 | 67 (6.2) | 20 (6.4) | 0.473 |
|  | Least deprived | 55 (10) | 15 (10.3) |  | 77 (9.3) | 24 (11) |  | 117 (10.9) | 39 (12.5) |  |
| Mortality |  | 191 (34.7) | 45 (31) | 0.431 | 256 (31) | 67 (30.6) | 0.935 | 358 (33.2) | 100 (31.9) | 0.683 |

Small numbers have been suppressed as part of disclosure control and indicated by x. Proportions in bold indicate statistical significance

Table C: Basic demography of admitted AKI-D patients in England grouped by day of admission in a propensity score matched cohort in each study period from 2009 to 2012.

|  |  | **2009-10** | | | **2010-11** | | | **2011-12** | | |
| --- | --- | --- | --- | --- | --- | --- | --- | --- | --- | --- |
|  |  | **Weekday** | **Weekend** | **p value** | **Weekday** | **Weekend** | **p value** | **Weekday** | **Weekend** | **p value** |
| Age group | <65 | 389 (28.7) | 87 (26) | 0.221 | 452 (29.3) | 119 (26.5) | 0.443 | 487 (28.7) | 147 (26.9) | 0.603 |
|  | 65 to 74 | 455 (33.6) | 132 (39.4) |  | 524 (34) | 153 (34.3) |  | 563 (33.2) | 186 (34.1) |  |
|  | 75 to 84 | 390 (28.8) | 92 (27.5) |  | 429 (27.8) | 140 (31.2) |  | 482 (28.4) | 167 (30.6) |  |
|  | >=85 | 120 (8.9) | 24 (7.2) |  | 138 (8.9) | 36 (8) |  | 163 (9.6) | 46 (8.4) |  |
| Gender | Male | 857 (63.3) | 203 (60.6) | 0.377 | 945 (61.2) | 280 (62.4) | 0.7 | 1071 (63.2) | 358 (65.6) | 0.331 |
| Ethnicity | White | 1065 (78.7) | 250 (74.6) | 0.26 | 1232 (79.8) | 341 (75.9) | 0.055 | 1349 (79.6) | 413 (75.6) | 0.22 |
|  | Black | 43 (3.2) | 7 (2.1) |  | 48 (3.1) | 10 (2.2) |  | 44 (2.6) | 13 (2.4) |  |
|  | Asian | 88 (6.5) | 26 (7.8) |  | 105 (6.8) | 38 (8.5) |  | 110 (6.5) | 36 (6.6) |  |
|  | Any other | 20 (1.5) | 8 (2.4) |  | 9 (0.6) | 8 (1.8) |  | 25 (1.5) | 12 (2.2) |  |
|  | Not known | 136 (10) | 44 (13.1) |  | 147 (9.5) | 52 (11.6) |  | 166 (9.8) | 72 (13.2) |  |
| Admission method | Elective | 30 (2.2) | x | 0.218 | 38 (2.5) | 7 (1.6) | 0.767 | 50 (2.9) | 13 (2.4) | 0.433 |
|  | Emergency | 1229 (90.8) | 298 (89) |  | 1395 (90.4) | 411 (91.5) |  | 1562 (92.2) | 496 (90.8) |  |
|  | Transfer | 88 (6.5) | 32 (9.6) |  | 107 (6.9) | 30 (6.7) |  | 80 (4.7) | 36 (6.6) |  |
| AKI in codes | primary | 704 (52) | 155 (46.3) | 0.17 | **772 (50)** | **188 (41.9)** | **0.006** | 773 (45.6) | 249 (45.6) | 0.88 |
|  | Secondary | 199 (14.7) | 54 (16.1) |  | **209 (13.5)** | **79 (17.6)** |  | 250 (14.7) | 85 (15.6) |  |
|  | Other | 451 (33.3) | 126 (37.6) |  | **562 (36.4)** | **182 (40.5)** |  | 672 (39.6) | 212 (38.8) |  |
| Comorbidities | Acute myocardial infarction | **194 (14.3)** | **64 (19.1)** | **0.034** | 228 (14.8) | 79 (17.6) | 0.158 | 259 (15.3) | 76 (13.9) | 0.49 |
|  | Cerebrovascular accident | 34 (2.5) | 9 (2.7) | 0.847 | 49 (3.2) | 11 (2.4) | 0.531 | 60 (3.5) | 19 (3.5) | 1 |
|  | Congestive cardiac failure | 289 (21.3) | 71 (21.2) | 1 | 362 (23.5) | 110 (24.5) | 0.659 | 420 (24.8) | 143 (26.2) | 0.533 |
|  | Connective tissue disorder | 69 (5.1) | 14 (4.2) | 0.573 | 88 (5.7) | 23 (5.1) | 0.726 | **95 (5.6)** | **16 (2.9)** | **0.012** |
|  | Kidney disease | 1170 (86.4) | 294 (87.8) | 0.59 | 1302 (84.4) | 378 (84.2) | 0.941 | 1399 (82.5) | 459 (84.1) | 0.433 |
|  | Peptic ulcer | 43 (3.2) | **x** |  | 52 (3.4) | 17 (3.8) | 0.661 | 58 (3.4) | 14 (2.6) | 0.402 |
|  | Peripheral vascular disease | 132 (9.7( | 38 (11.3) | 0.417 | 148 (9.6) | 43 (9.6) | 1 | 189 (11.2) | 52 (9.5) | 0.303 |
|  | Lung disease | 174 (12.9) | 42 (12.5) | 0.927 | 245 (15.9) | 70 (15.6) | 0.941 | 280 (16.5) | 82 (15) | 0.423 |
|  | Paraplegia | 15 (1.1) | x | 0.572 | 17 (1.1) | x | 1 | 35 (2.1) | 8 (1.5) | 0.474 |
|  | Diabetes mellitus | 475 (35.1) | 122 (36.4) | 0.655 | 557 (36.1) | 153 (34.1) | 0.467 | 561 (33.1) | 179 (32.8) | 0.917 |
|  | Liver disease | 82 (6.1) | 23 (6.9) | 0.613 | 141 (9.1) | 43 (9.6) | 0.781 | 167 (9.9) | 54 (9.9) | 1 |
|  | Malignancy | 182 (13.4) | 50 (14.9) | 0.479 | 250 (16.2) | 68 (15.1) | 0.609 | 252 (14.9) | 77 (14.1) | 0.728 |
| Deprivation | Most deprived | 63 (4.7) | 12 (3.6) | 0.989 | 108 (7) | 32 (7.1) | 0.642 | 68 (4) | 16 (2.9) | 0.08 |
|  | Least deprived | 166 (12.3) | 38 (11.3) |  | 184 (11.9) | 52 (11.6) |  | 202 (11.9) | 65 (11.9) |  |
| Mortality |  | 509 (37.6) | 123 (36.7) | 0.801 | **564 (36.6)** | **198 (44.1)** | **0.004** | 660 (38.9) | 232 (42.5) | 0.145 |

Small numbers have been suppressed as part of disclosure control and indicated by x. Proportions in bold indicate statistical significance

Table D: Basic demography of admitted AKI-D patients in England grouped by day of admission in a propensity score matched cohort in each study period from 2012 to 2015.

|  |  | **2012-13** | | | **2013-14** | | | **2014-15** | | |
| --- | --- | --- | --- | --- | --- | --- | --- | --- | --- | --- |
|  |  | **Weekday** | **Weekend** | **p value** | **Weekday** | **Weekend** | **p value** | **Weekday** | **Weekend** | **p value** |
| Age group | <65 | 580 (29.1) | 171 (29.5) | 0.285 | 698 (30.5) | 201 (30.5) | 0.961 | 826 (32.7) | 250 (31.6) | 0.85 |
|  | 65 to 74 | 709 (35.6) | 184 (31.7) |  | 760 (33.2) | 214 (32.4) |  | 838 (33.2) | 258 (32.7) |  |
|  | 75 to 84 | 548 (27.5) | 171 (29.5) |  | 637 (27.8) | 185 (28) |  | 658 (26) | 218 (27.6) |  |
|  | >=85 | 156 (7.8) | 54 (9.3) |  | 195 (8.5) | 60 (9.1) |  | 204 (8.1) | 64 (8.1) |  |
| Gender | Male | 1239 (62.2) | 371 (64) | 0.436 | 1435 (62.7) | 412 (62.4 |  | 1605 (63.5) | 508 (64.3) | 0.703 |
| Ethnicity | White | 1558 (78.2) | 434 (74.8) | 0.521 | 1762 (76.9) | 510 (77.3) | 0.473 | 1929 (76.4) | 626 (79.2) | 0.716 |
|  | Black | 64 (3.2) | 23 (4) |  | 60 (2.6) | 21 (3.2) |  | 80 (3.2) | 24 (3) |  |
|  | Asian | 127 (6.4) | 36 (6.2) |  | 147 (6.4) | 41 (6.2) |  | 163 (6.5) | 43 (5.4) |  |
|  | Any other | 34 (1.7) | 12 (2.1) |  | 56 (2.4) | 10 (1.5) |  | 42 (1.7) | 14 (1.8) |  |
|  | Not known | 206 (10.3) | 74 (12.8) |  | 257 (11.2) | 78 11.8) |  | 309 (12.2) | 82 (10.4) |  |
| Admission method | Elective | 47 (2.4) | 12 (2.1) | 0.574 | 57 (2.5) | 12 (1.8) | 0.449 | 36 (1.4) | 13 (1.6) | 0.821 |
|  | Emergency | 1823 (91.5) | 523 (90.2) |  | 1828 (79.8) | 546 (82.7) |  | 1867 (73.9) | 589 (74.6) |  |
|  | Transfer | 119 (6) | 44 (7.6) |  | 125 (5.5) | 35 (5.3) |  | 115 (4.6) | 35 (4.4) |  |
|  | Not known | x | x |  | 274 (12) | 67 (10.2) |  | 504 (20) | 150 (19) |  |
| AKI in codes | Primary | **918 (46.1)** | **263 (45.3)** | **0.035** | 929 (40.60 | 273 (41.4) | 0.732 | 938 (37.1) | 302 (38.2) | 0.334 |
|  | Secondary | **294 (14.8)** | **64 (11)** |  | 351 (15.3) | 93 (14.1) |  | 407 (16.1) | 110 (13.9) |  |
|  | Other | **781 (39.2)** | **253 (43.6)** |  | 1010 (44.1) | 294 (44.5) |  | 1181 (46.8) | 378 (47.8) |  |
| Comorbidities | Acute myocardial infarction | 267 (13.4) | 91 (15.7) | 0.173 | 330 (14.4) | 87 (13.2) | 0.447 | 304 (12) | 106 (13.4) | 0.322 |
|  | Cerebrovascular accident | 60 (3) | 21 (3.6) | 0.499 | 80 (3.5) | 24 (3.6) | 0.905 | 84 (3.3) | 27 (3.4) | 0.91 |
|  | Congestive cardiac failure | 514 (25.8) | 146 (25.2) | 0.787 | 620 (27.1) | 186 (28.2) | 0.586 | 670 (26.5) | 214 (27.1) | 0.747 |
|  | Connective tissue disorder | 104 (5.2) | 28 (4.8) | 0.75 | **91 (4)** | **46 (7)** | **0.002** | 139 (5.5) | 43 (5.4) | 1 |
|  | Dementia | x | x | x | 9 (0.4) | x | 0.737 | 6 (0.2) | x | 1 |
|  | Kidney disease | 1672 (83.9) | 487 (84) | 1 | 1793 (78.3) | 529 (80.2) | 0.331 | 1907 (75.5) | 586 (74.2) | 0.451 |
|  | Peptic ulcer | 64 (3.2) | 17 (2.9) | 0.789 | 73 (3.2) | 20 (3) | 0.9 | 67 (2.7) | 25 (3.2) | 0.457 |
|  | Peripheral vascular disease | 196 (9.8) | 72 (12.4) | 0.076 | 240 (10.5) | 67 (10.2) | 0.885 | 263 (10.4) | 68 (8.6) | 0.153 |
|  | Lung disease | 332 (16.7) | 80 (13.8) | 0.108 | 394 (17.2) | 106 (16.1) | 0.517 | 421 (16.7) | 145 (18.4) | 0.279 |
|  | Paraplegia | 25 (1.3) | 10 (1.7) | 0.415 | 29 (1.3) | 8 (1.2) | 1 | 29 (1.1) | 12 (1.5) | 0.46 |
|  | Diabetes mellitus | 681 (34.2) | 186 (32.1) | 0.369 | 773 (33.8) | 237 (35.9) | 0.306 | 861 (34.1) | 282 (35.7) | 0.415 |
|  | Liver disease | 198 (9.9) | 60 (10.3) | 0.754 | 229 (10) | 65 (9.8) | 0.941 | 265 (10.5) | 68 (8.6) | 0.136 |
|  | Malignancy | 320 (16.1) | 93 (16) | 1 | 328 (14.3) | 86 (13) | 0.445 | 338 (13.4) | 115 (14.6) | 0.406 |
| Deprivation | Most deprived | 97 (4.9) | 31 (5.3) | 0.37 | 140 (6.1) | 40 (6.1) | 0.956 | 148 (5.9) | 48 (6.1) | 0.114 |
|  | Least deprived | 234 (11.7) | 59 (10.2) |  | 244 (10.7) | 68 (10.3) |  | 294 (11.6) | 71 (9) |  |
| Mortality |  | 760 (38.1) | 244 (42.1) | 0.091 | 916 (40) | 257 (38.9) | 0.652 | 1010 (40) | 306 (38.7) | 0.56 |

Small numbers have been suppressed as part of disclosure control and indicated by x. Proportions in bold indicate statistical significance
