# Supplementary material for: Effect of weekend admission on mortality associated with severe acute kidney injury in England: A propensity score matched, population-based study
Source: PLoS One. 2017 Oct 10;12(10):e0186048. doi: 10.1371/journal.pone.0186048 (PMC5634642; doi:10.1371/journal.pone.0186048)
Supplement: S1 Table — (DOCX) [file pone.0186048.s003.docx]

S1 table: Effect of weekend admission on mortality after exclusion of patients with unknown ethnicity.

| Admission day | Univariate | Multivariable**^§^** | In-center nephrology services | Univariate | Multivariable**^§^** |
| --- | --- | --- | --- | --- | --- |
| Weekday | 1 (Ref) | 1 (Ref) | No | 1 (Ref) | 1 (Ref) |
| Weekend | 1.05 (0.97, 1.13) | 1.01 (0.93, 1.09) | Yes | **0.5 (0.46, 0.53)** | **0.58 (0.54, 0.63)** |

**^§^** Adjusted for age group, gender, ethnicity, AKI in diagnoses codes, deprivation, admission methods, CCI. Odds ratios and confidence intervals in

bold indicate statistical significance.
